# A Case of Acute Yellow Atrophy of the Liver in a Child
1Read at a meeting of the Society on January 10th, 1906.


**Published:** 1906-03

**Authors:** Bertram M. H. Rogers

**Affiliations:** Physician to the Royal Hospital for Sick Children and Women, Bristol


### A CASE OF ACUTE YELLOW ATROPHY OF THE LIVER IN A CHILD.[1]

BY

BERTRAM M. H. ROGERS, B.A., M.D., B.Ch.Oxon.,

*Physician to the Royal Hospital for Sick Children and Women, Bristol.*

---

THIS case differs in few particulars from the accounts given in the text-books of this disease. It is undoubtedly of rare occurrence even in adults. We have no records of a similar case at the Children's Hospital, and I do not find one has been reported before the Society for the Study of Diseases in Children during the last five years. It has been said that in one of our large London hospitals one is seen in 500,000 cases.

The patient was a boy of 4 years of age, and from the history given by the parents appears to have been a healthy lad till a short time before admission. He was the third of three living children, two others having died in infancy from wasting. Six months before admission he had had measles, but had recovered satisfactorily. Three months before admission as an in-patient he had been attending the hospital as an out-patient "with cough, weakness, and a yellowish tinge in the skin." From this he is said to have recovered, for he was "quite well till about a week ago, when he was noticed to be distinctly yellow, and was very sick, especially at night." He complained considerably of abdominal pain, and his parents said his motions were pale and the water very yellow. On June 1st he was delirious, and complained of headache. He seemed feverish, we were told, but we have no record of a medical man having seen him. The skin was irritable, but there was no rash. We could get no history of poisonous food, and no similar case had been heard of in the neighbourhood. There had been no hemorrhages, rash, sore throat, or running from the eyes.

[1] Read at a meeting of the Society on January 10th, 1906.

The patient was admitted to the Children's Hospital on June 2nd, and he was then obviously very ill, being in a state of noisy delirium. His temperature varied in the first twenty-four hours between 97° and 96°. The skin was intensely jaundiced, very dry, and evidently very irritable, to judge from the numerous marks of scratching. His lips and teeth were covered with sordes. The urine was deeply coloured, contained no albumin, was acid, and the sp. gr. was 1015. The only other symptom was a transient squint. During the second day his temperature rose to 103.6°, and at death on June 4th it was found to be 108°.

Examination showed that the liver extended to two finger-breadths below the costal margin in the nipple line, and the spleen could be felt just below the ribs, both organs feeling firm to the touch. The *post mortem* was made by Dr. Brickdale on June 5th. The organs were all deeply stained with bile, the heart and lungs appearing healthy. The liver weighed 17 oz., this being probably about the normal weight of the organ for a boy of this age. The substance was of a dull red colour, but there were no hemorrhages anywhere. The gall duct was patent, for bile from the gall-bladder could be pressed into the intestine. The spleen weighed 4½ oz., possibly rather larger in size than usual; its substance was soft. The thymus was large, and adherent to the apex of the right lung and pericardium; it was dull red in colour, and in a cavity was a not inconsiderable amount of bright yellow debris, probably the remains of an old abscess. The *post-mortem* notes say that there was some pus in the pelvis of one kidney, but there is no note of a microscopic examination of the fluid, or any reference to a diseased condition of the kidney. I am inclined, speaking from memory, to say that the fluid was only turbid urine.

I have called this case one of acute yellow atrophy, because the clinical symptoms agree better with those of that disease than with any other form of icterus gravis. There was, it is true, no atrophy of the liver; but this can be explained, I believe, by the rapidly fatal termination preventing the shrinking that is often or generally seen in these cases.

What the actual cause of the sudden intense jaundice and fatal result came from has yet to be explained. There must have been some very virulent poison at work, possibly for some time in a mild form, acting on the liver cells, and then either a sudden exacerbation or increase of poison, which rapidly destroyed the liver cells.

That the liver substance suffered a rapid disintegration is evident from the microscopic slides made of the liver. In them no trace of liver cells can be seen. The liver looks as if nothing but a fine reticulum of supporting tissue had been left, "the skeleton of the lobules formed of the fibrillar vascular connective tissue containing a few nuclei." (Rolleston.)

The specimen of urine obtained was unfortunately not examined for leucin and tyrosin. Their presence is not diagnostic, nor does their absence exclude the disease.

---

### NOTES ON A CASE OF SPLENOMEGALIC BILIARY CIRRHOSIS, IN A BOY AGED SIX YEARS.[1]

BY

EDWARD CECIL WILLIAMS, M.B.Cantab.,

